# Dichloridobis[2-(2-fur­yl)-1-(2-furylmeth­yl)-1*H*-benzimidazole-κ*N* ^3^]cadmium(II)

**DOI:** 10.1107/S1600536810034409

**Published:** 2010-09-04

**Authors:** Xia Wang, Yu-Xian Li, Yan-Ju Liu, Huai-Xia Yang, Cong-Cong Zhang

**Affiliations:** a Pharmacy College, Henan University of Traditional Chinese Medicine, Zhengzhou 450008, People’s Republic of China

## Abstract

In the title complex, [CdCl_2_(C_16_H_12_N_2_O_2_)_2_], the Cd^II^ ion exhibits site symmetry 2. It shows a distorted tetra­hedral coordination defined by two N atoms from symmetry-related 2-(2-fur­yl)-1-(2-furylmeth­yl)-1*H*-benzimidazole ligands and by two symmetry-related Cl atoms. Intra­molecular C—H⋯O hydrogen bonds stabilize the mol­ecular configuration. Adjacent mol­ecules are linked through C—H⋯Cl hydrogen bonds into a network structure.

## Related literature

For background to benzimidazoles, see: Shen & Yuan (2006 ▶); Yang *et al.* (2008 ▶). For background to Cd^II^ complexes, see: Meng *et al.* (2004 ▶); Yang *et al.* (2010 ▶).
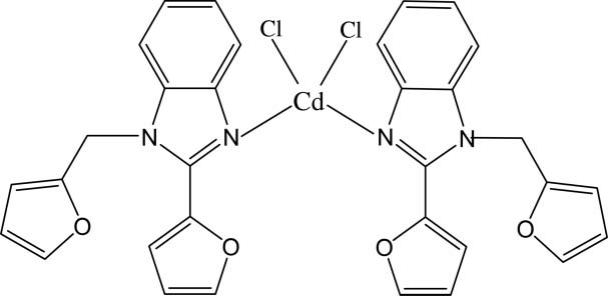


## Experimental

### 

#### Crystal data

[CdCl_2_(C_16_H_12_N_2_O_2_)_2_]
*M* *_r_* = 711.85
Monoclinic, 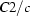

*a* = 18.397 (4) Å
*b* = 10.451 (2) Å
*c* = 17.470 (3) Å
β = 116.72 (3)°
*V* = 3000.2 (13) Å^3^
*Z* = 4
Mo *K*α radiation
μ = 0.95 mm^−1^
*T* = 293 K
0.21 × 0.19 × 0.16 mm

#### Data collection

Rigaku Saturn diffractometer
Absorption correction: multi-scan (*REQAB*; Jacobson, 1998 ▶) *T*_min_ = 0.825, *T*_max_ = 0.863
10628 measured reflections
2953 independent reflections
2565 reflections with *I* > 2σ(*I*)
*R*_int_ = 0.039

#### Refinement

*R*[*F* ^2^ > 2σ(*F* ^2^)] = 0.048
*wR*(*F* ^2^) = 0.110
*S* = 1.10
2953 reflections
195 parameters
H-atom parameters constrained
Δρ_max_ = 0.34 e Å^−3^
Δρ_min_ = −0.47 e Å^−3^

### 

Data collection: *CrystalClear* (Rigaku/MSC, 2006 ▶); cell refinement: *CrystalClear*; data reduction: *CrystalClear*; program(s) used to solve structure: *SHELXS97* (Sheldrick, 2008 ▶); program(s) used to refine structure: *SHELXL97* (Sheldrick, 2008 ▶); molecular graphics: *XP* in *SHELXTL* (Sheldrick, 2008 ▶); software used to prepare material for publication: *SHELXTL*.

## Supplementary Material

Crystal structure: contains datablocks global, I. DOI: 10.1107/S1600536810034409/wm2393sup1.cif

Structure factors: contains datablocks I. DOI: 10.1107/S1600536810034409/wm2393Isup2.hkl

Additional supplementary materials:  crystallographic information; 3D view; checkCIF report

## Figures and Tables

**Table:** 

Cd1—N1	2.252 (3)
Cd1—Cl1	2.4513 (12)

**Table:** 

N1^i^—Cd1—N1	118.17 (15)
N1^i^—Cd1—Cl1	109.08 (8)
N1—Cd1—Cl1	106.27 (8)
Cl1^i^—Cd1—Cl1	107.57 (6)

Symmetry code: (i) 
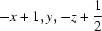
.

**Table 2:** Hydrogen-bond geometry (Å, °)

*D*—H⋯*A*	*D*—H	H⋯*A*	*D*⋯*A*	*D*—H⋯*A*
C5—H5*A*⋯Cl1^ii^	0.93	2.82	3.694 (5)	156
C9—H9*A*⋯O2	0.93	2.49	3.256 (6)	140

Symmetry code: (ii) 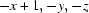.

**Fig. 1.**
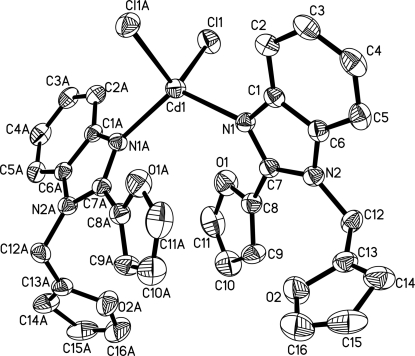
View of the title complex, showing the labelled atoms and displacement ellipsoids at the 30% probability level. H atoms were omitted for clarity. [Symmetry code A: -x+1, y, -z+0.5.]

## supplementary crystallographic information

### Comment

Benzimidazole and its derivatives have attracted interest because of their biological activities as well as their abilities to bind to different metal ions (Shen & Yuan, 2006; Yang *et al.*, 2008). The Cd^II^ ion is a good model atom to construct complexes owing to its property to form bonds with different donors simultaneously, and to its various coordination modes (Meng *et al.*, 2004; Yang *et al.*, 2010). In this work, we describe the synthesis and structure of the title complex, [CdCl_2_(C_16_H_12_N_2_O_2_)_2_], (I).

In the structure of (I),  two 2-(furan-2-yl)-1-(furan-2-yl-methyl)-1*H*-1,3-benzimidazole ligands and two Cl atoms coordinate to the Cd^II^ ion which is located on a twofold rotation axis. As expected, the Cd—Cl bond length is slightly longer than the Cd—N bond length. The environment around the Cd^II^ ion can be best described as distorted tetrahedral (Fig.1).

Intramolecular C—H···O hydrogen bonds stabilize the molecular configuration and C—H···Cl hydrogen bonds between adjacent molecules consolidate the crystal packing.

### Experimental

The ligand 2-(furan-2-yl)-1-(furan-2-yl-methyl)-1*H*-1,3-benzimidazole (0.04 mmol) in methanol (7 ml) was added dropwise to a methanol solution (5 ml) of CdCl_2_ (0.02 mmol). The resulting solution was allowed to stand at room temperature. After one week colorless crystals with good quality were obtained from the filtrate and dried in air.

### Refinement

H atoms were positioned geometrically and refined as riding atoms, with C-H = 0.93 (aromatic) and 0.97 (CH_2_) Å and with U_iso_(H) = 1.2 U_eq_(C).

### Crystal data

**Table:**

[CdCl_2_(C_16_H_12_N_2_O_2_)_2_]	*F*(000) = 1432
*M**_r_* = 711.85	*D*_x_ = 1.576 Mg m^−^^3^
Monoclinic, *C*2/*c*	Mo *K*α radiation, λ = 0.71073 Å
Hall symbol: -C 2yc	Cell parameters from 4133 reflections
*a* = 18.397 (4) Å	θ = 2.3–27.9°
*b* = 10.451 (2) Å	µ = 0.95 mm^−^^1^
*c* = 17.470 (3) Å	*T* = 293 K
β = 116.72 (3)°	Prism, colorless
*V* = 3000.2 (13) Å^3^	0.21 × 0.19 × 0.16 mm
*Z* = 4	

### Data collection

**Table:**

Rigaku Saturn diffractometer	2953 independent reflections
Radiation source: fine-focus sealed tube	2565 reflections with *I* > 2σ(*I*)
graphite	*R*_int_ = 0.039
Detector resolution: 28.5714 pixels mm^-1^	θ_max_ = 26.0°, θ_min_ = 2.3°
ω scans	*h* = −22→22
Absorption correction: multi-scan (*REQAB*; Jacobson, 1998)	*k* = −12→10
*T*_min_ = 0.825, *T*_max_ = 0.863	*l* = −21→21
10628 measured reflections	

### Refinement

**Table:**

Refinement on *F*^2^	Primary atom site location: structure-invariant direct methods
Least-squares matrix: full	Secondary atom site location: difference Fourier map
*R*[*F*^2^ > 2σ(*F*^2^)] = 0.048	Hydrogen site location: inferred from neighbouring sites
*wR*(*F*^2^) = 0.110	H-atom parameters constrained
*S* = 1.10	*w* = 1/[σ^2^(*F*_o_^2^) + (0.0536*P*)^2^] where *P* = (*F*_o_^2^ + 2*F*_c_^2^)/3
2953 reflections	(Δ/σ)_max_ < 0.001
195 parameters	Δρ_max_ = 0.34 e Å^−^^3^
0 restraints	Δρ_min_ = −0.47 e Å^−^^3^

### Special details

**Table:**

Geometry. All esds (except the esd in the dihedral angle between two l.s. planes) are estimated using the full covariance matrix. The cell esds are taken into account individually in the estimation of esds in distances, angles and torsion angles; correlations between esds in cell parameters are only used when they are defined by crystal symmetry. An approximate (isotropic) treatment of cell esds is used for estimating esds involving l.s. planes.
Refinement. Refinement of F^2^ against ALL reflections. The weighted R-factor wR and goodness of fit S are based on F^2^, conventional R-factors R are based on F, with F set to zero for negative F^2^. The threshold expression of F^2^ > 2sigma(F^2^) is used only for calculating R-factors(gt) etc. and is not relevant to the choice of reflections for refinement. R-factors based on F^2^ are statistically about twice as large as those based on F, and R- factors based on ALL data will be even larger.

### Fractional atomic coordinates and isotropic or equivalent isotropic displacement parameters (Å^2^)

**Table:**

	*x*	*y*	*z*	*U*_iso_*/*U*_eq_	
Cd1	0.5000	−0.08924 (3)	0.2500	0.04422 (17)	
Cl1	0.61483 (7)	−0.22781 (11)	0.27047 (8)	0.0670 (3)	
O1	0.6047 (2)	0.1489 (3)	0.2577 (2)	0.0742 (9)	
O2	0.3968 (2)	0.4328 (3)	0.0331 (2)	0.0822 (11)	
N1	0.46860 (17)	0.0215 (3)	0.12856 (19)	0.0401 (7)	
N2	0.46152 (18)	0.1695 (3)	0.0334 (2)	0.0439 (7)	
C1	0.4133 (2)	−0.0189 (3)	0.0476 (2)	0.0400 (9)	
C2	0.3666 (2)	−0.1304 (4)	0.0213 (3)	0.0487 (10)	
H2A	0.3693	−0.1927	0.0605	0.058*	
C3	0.3166 (2)	−0.1443 (4)	−0.0649 (3)	0.0552 (11)	
H3A	0.2846	−0.2173	−0.0842	0.066*	
C4	0.3126 (3)	−0.0521 (5)	−0.1240 (3)	0.0613 (12)	
H4A	0.2782	−0.0653	−0.1818	0.074*	
C5	0.3582 (3)	0.0584 (4)	−0.0994 (3)	0.0541 (11)	
H5A	0.3556	0.1202	−0.1388	0.065*	
C6	0.4083 (2)	0.0723 (3)	−0.0121 (3)	0.0426 (9)	
C7	0.4950 (2)	0.1349 (3)	0.1171 (2)	0.0407 (8)	
C8	0.5529 (2)	0.2124 (4)	0.1863 (3)	0.0476 (9)	
C9	0.5623 (3)	0.3387 (4)	0.1971 (3)	0.0574 (11)	
H9A	0.5330	0.4016	0.1576	0.069*	
C10	0.6258 (3)	0.3579 (6)	0.2805 (4)	0.0835 (17)	
H10A	0.6469	0.4363	0.3060	0.100*	
C11	0.6496 (3)	0.2440 (7)	0.3154 (3)	0.0882 (17)	
H11A	0.6905	0.2294	0.3705	0.106*	
C12	0.4755 (2)	0.2850 (4)	−0.0060 (3)	0.0520 (10)	
H12A	0.4817	0.2609	−0.0564	0.062*	
H12B	0.5257	0.3254	0.0342	0.062*	
C13	0.4077 (3)	0.3782 (4)	−0.0311 (3)	0.0532 (11)	
C14	0.3522 (3)	0.4196 (4)	−0.1061 (3)	0.0719 (14)	
H14A	0.3473	0.3972	−0.1597	0.086*	
C15	0.3012 (3)	0.5054 (5)	−0.0888 (4)	0.0888 (19)	
H15A	0.2564	0.5498	−0.1290	0.107*	
C16	0.3296 (4)	0.5097 (5)	−0.0057 (5)	0.097 (2)	
H16A	0.3073	0.5581	0.0232	0.117*	

### Atomic displacement parameters (Å^2^)

**Table:**

	*U*^11^	*U*^22^	*U*^33^	*U*^12^	*U*^13^	*U*^23^
Cd1	0.0472 (3)	0.0419 (3)	0.0376 (2)	0.000	0.01378 (19)	0.000
Cl1	0.0685 (7)	0.0712 (8)	0.0582 (7)	0.0256 (6)	0.0256 (6)	0.0060 (6)
O1	0.076 (2)	0.082 (2)	0.057 (2)	0.0048 (19)	0.0234 (18)	0.0035 (18)
O2	0.103 (3)	0.079 (2)	0.077 (2)	0.046 (2)	0.052 (2)	0.0202 (18)
N1	0.0410 (17)	0.0379 (18)	0.0373 (17)	0.0028 (14)	0.0141 (14)	0.0035 (13)
N2	0.0451 (18)	0.0402 (18)	0.0467 (19)	0.0071 (15)	0.0209 (15)	0.0041 (15)
C1	0.037 (2)	0.042 (2)	0.038 (2)	0.0097 (16)	0.0150 (17)	0.0002 (16)
C2	0.044 (2)	0.047 (2)	0.053 (2)	0.0025 (18)	0.0194 (19)	−0.0037 (19)
C3	0.046 (2)	0.061 (3)	0.050 (3)	0.001 (2)	0.013 (2)	−0.017 (2)
C4	0.053 (3)	0.076 (3)	0.038 (2)	0.012 (2)	0.006 (2)	−0.010 (2)
C5	0.053 (3)	0.065 (3)	0.042 (2)	0.018 (2)	0.019 (2)	0.008 (2)
C6	0.040 (2)	0.045 (2)	0.042 (2)	0.0131 (17)	0.0170 (17)	0.0022 (17)
C7	0.040 (2)	0.039 (2)	0.041 (2)	0.0060 (17)	0.0168 (17)	0.0031 (16)
C8	0.043 (2)	0.052 (2)	0.049 (2)	−0.0006 (19)	0.0220 (19)	0.0009 (19)
C9	0.062 (3)	0.040 (2)	0.063 (3)	−0.012 (2)	0.021 (2)	−0.003 (2)
C10	0.080 (4)	0.085 (4)	0.084 (4)	−0.045 (3)	0.035 (3)	−0.037 (3)
C11	0.060 (3)	0.134 (5)	0.056 (3)	−0.006 (4)	0.013 (3)	−0.018 (4)
C12	0.057 (3)	0.049 (2)	0.057 (3)	0.0098 (19)	0.033 (2)	0.0160 (19)
C13	0.059 (3)	0.040 (2)	0.065 (3)	0.0088 (19)	0.031 (2)	0.009 (2)
C14	0.079 (4)	0.052 (3)	0.066 (3)	0.014 (2)	0.016 (3)	0.002 (2)
C15	0.074 (4)	0.051 (3)	0.111 (5)	0.021 (3)	0.015 (4)	0.008 (3)
C16	0.099 (5)	0.077 (4)	0.125 (6)	0.045 (3)	0.059 (4)	0.012 (4)

### Geometric parameters (Å, °)

**Table:**

Cd1—N1^i^	2.252 (3)	C4—H4A	0.9300
Cd1—N1	2.252 (3)	C5—C6	1.389 (5)
Cd1—Cl1^i^	2.4513 (12)	C5—H5A	0.9300
Cd1—Cl1	2.4513 (12)	C7—C8	1.447 (5)
O1—C8	1.354 (5)	C8—C9	1.334 (5)
O1—C11	1.392 (6)	C9—C10	1.414 (7)
O2—C13	1.352 (5)	C9—H9A	0.9300
O2—C16	1.372 (6)	C10—C11	1.319 (7)
N1—C7	1.330 (5)	C10—H10A	0.9300
N1—C1	1.386 (4)	C11—H11A	0.9300
N2—C7	1.356 (5)	C12—C13	1.484 (5)
N2—C6	1.387 (5)	C12—H12A	0.9700
N2—C12	1.469 (5)	C12—H12B	0.9700
C1—C6	1.386 (5)	C13—C14	1.320 (6)
C1—C2	1.396 (5)	C14—C15	1.424 (7)
C2—C3	1.374 (6)	C14—H14A	0.9300
C2—H2A	0.9300	C15—C16	1.305 (8)
C3—C4	1.390 (6)	C15—H15A	0.9300
C3—H3A	0.9300	C16—H16A	0.9300
C4—C5	1.378 (6)		
			
N1^i^—Cd1—N1	118.17 (15)	N1—C7—C8	123.6 (3)
N1^i^—Cd1—Cl1^i^	106.27 (8)	N2—C7—C8	124.0 (3)
N1—Cd1—Cl1^i^	109.08 (8)	C9—C8—O1	111.2 (4)
N1^i^—Cd1—Cl1	109.08 (8)	C9—C8—C7	132.2 (4)
N1—Cd1—Cl1	106.27 (8)	O1—C8—C7	116.4 (4)
Cl1^i^—Cd1—Cl1	107.57 (6)	C8—C9—C10	106.3 (4)
C8—O1—C11	105.1 (4)	C8—C9—H9A	126.8
C13—O2—C16	105.9 (4)	C10—C9—H9A	126.8
C7—N1—C1	105.5 (3)	C11—C10—C9	107.3 (4)
C7—N1—Cd1	130.1 (2)	C11—C10—H10A	126.4
C1—N1—Cd1	124.4 (2)	C9—C10—H10A	126.4
C7—N2—C6	106.5 (3)	C10—C11—O1	110.1 (5)
C7—N2—C12	129.4 (3)	C10—C11—H11A	125.0
C6—N2—C12	124.1 (3)	O1—C11—H11A	125.0
N1—C1—C6	109.2 (3)	N2—C12—C13	112.1 (3)
N1—C1—C2	130.6 (4)	N2—C12—H12A	109.2
C6—C1—C2	120.2 (4)	C13—C12—H12A	109.2
C3—C2—C1	117.3 (4)	N2—C12—H12B	109.2
C3—C2—H2A	121.4	C13—C12—H12B	109.2
C1—C2—H2A	121.4	H12A—C12—H12B	107.9
C2—C3—C4	121.8 (4)	C14—C13—O2	110.3 (4)
C2—C3—H3A	119.1	C14—C13—C12	133.0 (5)
C4—C3—H3A	119.1	O2—C13—C12	116.7 (4)
C3—C4—C5	121.8 (4)	C13—C14—C15	106.7 (5)
C3—C4—H4A	119.1	C13—C14—H14A	126.7
C5—C4—H4A	119.1	C15—C14—H14A	126.7
C4—C5—C6	116.2 (4)	C16—C15—C14	106.5 (5)
C4—C5—H5A	121.9	C16—C15—H15A	126.7
C6—C5—H5A	121.9	C14—C15—H15A	126.7
N2—C6—C1	106.3 (3)	C15—C16—O2	110.5 (5)
N2—C6—C5	130.9 (4)	C15—C16—H16A	124.7
C1—C6—C5	122.7 (4)	O2—C16—H16A	124.7
N1—C7—N2	112.4 (3)		
			
N1^i^—Cd1—N1—C7	31.3 (3)	Cd1—N1—C7—C8	−0.1 (5)
Cl1^i^—Cd1—N1—C7	152.8 (3)	C6—N2—C7—N1	1.3 (4)
Cl1—Cd1—N1—C7	−91.5 (3)	C12—N2—C7—N1	−178.8 (3)
N1^i^—Cd1—N1—C1	−147.1 (3)	C6—N2—C7—C8	−178.6 (4)
Cl1^i^—Cd1—N1—C1	−25.7 (3)	C12—N2—C7—C8	1.3 (6)
Cl1—Cd1—N1—C1	90.0 (3)	C11—O1—C8—C9	−0.9 (5)
C7—N1—C1—C6	0.8 (4)	C11—O1—C8—C7	−176.4 (4)
Cd1—N1—C1—C6	179.6 (2)	N1—C7—C8—C9	−147.9 (5)
C7—N1—C1—C2	−179.6 (4)	N2—C7—C8—C9	32.1 (7)
Cd1—N1—C1—C2	−0.8 (5)	N1—C7—C8—O1	26.4 (6)
N1—C1—C2—C3	−179.7 (4)	N2—C7—C8—O1	−153.6 (4)
C6—C1—C2—C3	−0.2 (5)	O1—C8—C9—C10	1.3 (5)
C1—C2—C3—C4	0.4 (6)	C7—C8—C9—C10	175.8 (5)
C2—C3—C4—C5	−0.3 (7)	C8—C9—C10—C11	−1.2 (6)
C3—C4—C5—C6	0.0 (6)	C9—C10—C11—O1	0.6 (7)
C7—N2—C6—C1	−0.7 (4)	C8—O1—C11—C10	0.1 (6)
C12—N2—C6—C1	179.4 (3)	C7—N2—C12—C13	−103.6 (5)
C7—N2—C6—C5	179.8 (4)	C6—N2—C12—C13	76.2 (5)
C12—N2—C6—C5	−0.1 (6)	C16—O2—C13—C14	1.3 (6)
N1—C1—C6—N2	0.0 (4)	C16—O2—C13—C12	−177.5 (4)
C2—C1—C6—N2	−179.7 (3)	N2—C12—C13—C14	−112.3 (6)
N1—C1—C6—C5	179.5 (3)	N2—C12—C13—O2	66.1 (5)
C2—C1—C6—C5	−0.1 (6)	O2—C13—C14—C15	−0.9 (6)
C4—C5—C6—N2	179.6 (4)	C12—C13—C14—C15	177.6 (5)
C4—C5—C6—C1	0.2 (6)	C13—C14—C15—C16	0.2 (7)
C1—N1—C7—N2	−1.3 (4)	C14—C15—C16—O2	0.6 (7)
Cd1—N1—C7—N2	180.0 (2)	C13—O2—C16—C15	−1.2 (7)
C1—N1—C7—C8	178.6 (4)		

Symmetry codes: (i) −*x*+1, *y*, −*z*+1/2.

### Hydrogen-bond geometry (Å, °)

**Table:**

*D*—H···*A*	*D*—H	H···*A*	*D*···*A*	*D*—H···*A*
C5—H5A···Cl1^ii^	0.93	2.82	3.694 (5)	156
C9—H9A···O2	0.93	2.49	3.256 (6)	140

Symmetry codes: (ii) −*x*+1, −*y*, −*z*.
